# Clinical factors associated with cerebral autoregulation in ischemic stroke related to small artery occlusion

**DOI:** 10.1186/s12883-022-02854-4

**Published:** 2022-09-22

**Authors:** Xiaohong Wu, Yanxia Zhou, Wenwei Qi, Yanxia Shen, Zhihao Lei, Kun Xiao, Pandeng Zhang, Jia Liu, Lijie Ren

**Affiliations:** 1 Department of Neurology, the First Affiliated Hospital of Shenzhen University, Shenzhen Second People’s Hospital, Shenzhen, 518035 China; 2 Tianjin Institute of Cardiology, the Second Hospital of Tianjin Medical University, Tianjin, 300211 China; 3 Department of Neurology, Pinghu Hospital of Shenzhen University, Shenzhen, China; 4 Shenzhen Institutes of Advanced Technology, Chinese Academy of Sciences, Shenzhen, China

**Keywords:** Cerebral autoregulation, Ischaemic stroke, Small artery occlusion

## Abstract

**Background:**

Existing data suggest that cerebral autoregulation (CA) varies among different subtypes of ischaemic stroke. CA is globally impaired in patients with small artery occlusion (SAO). However, the factors influencing CA impairment in patients remains to be elucidated.

**Methods:**

Stroke patients with SAO who underwent brain magnetic resonance imaging (MRI) were prospectively studied. Within 7 days after stroke onset, CA was recorded from the middle cerebral artery blood flow velocity and arterial blood pressure was simultaneously measured. Transfer function analysis was used to derive CA parameters, including gain and phase. Clinical characteristics, mean arterial pressure (MAP), biochemical findings, and cerebral small vessel disease (CSVD) markers on MRI were assessed in each patient. Factors associated with CA parameters were investigated. Univariate and multivariate linear regression analyses were conducted to determine the relationship between clinical factors and CA parameters.

**Results:**

Sixty-three SAO patients (age, 56.3 ± 9.9 years; 55 men) were enrolled in the study. In the multiple linear regression analysis, after controlling for relevant clinical factors, MAP on admission (ipsilateral *OR* = 0.99 and contralateral *OR* = 0.99, both *P* < 0.005) was a significant independent predictor of bilateral gain. MAP > 105 mmHg on admission (*OR* = 0.77, *P* = 0.019) was significantly associated with ipsilateral gain. Diabetes mellitus was a significant predictive factor for bilateral gain (ipsilateral *OR* = 1.32 and contralateral *OR* = 1.22, both *P* < 0.005). No correlations were found between CA parameters and CSVD characteristics.

**Conclusion:**

In SAO-related ischaemic stroke, patients with MAP > 105 mmHg on admission tended to have better ipsilateral CA. Diabetes mellitus appears to be an independent risk factor for CA impairment in patients with SAO-related stroke. CSVD may not be the main factor affecting bilateral CA in patients with SAO.

**Supplementary Information:**

The online version contains supplementary material available at 10.1186/s12883-022-02854-4.

## Background

Cerebral autoregulation (CA) is an internal protective mechanism that maintains cerebral blood flow (CBF) at a relatively constant level despite fluctuations in cerebral perfusion pressure or arterial blood pressure (ABP) [1]. Generally, CA remains relatively intact when the mean arterial pressure (MAP) ranges between 50 and 170 mmHg. This is a physiological mechanism that may involve myogenic, neural, metabolic, and endothelial regulation of cerebral vasculature in response to changes in pressure or in cerebral blood flow [1, 2].

Impaired CA is observed in ischaemic stroke and may be associated with worse outcomes [3–5]. Therefore, protection of CA is crucial. Small artery occlusion (SAO) and large artery atherosclerosis (LAA) are the two main etiological subtypes of stroke according to the Trial of Org 10,172 in Acute Stroke Treatment (TOAST) classification [6, 7]. Notably, each stroke subtype has a particular CA characteristic [8–10]. Stroke associated with SAO, also known as lacunae stroke, is diagnosed based on magnetic resonance imaging (MRI) evidence of a single, clinically relevant, acute ischaemic lesion ≤15 mm in diameter, in the deep white matter, basal ganglia, or brainstem-penetrating arteries, in the absence of any other pathogenesis in the large parent artery. Unlike LAA, which affects CA ipsilaterally, SAO is more likely to impair CA bilaterally [11].

To date, few studies have revealed factors that influence dysregulation of CA in stroke patients. One study, which enrolled 67 stroke patients regardless of their stroke subtypes, demonstrated that increasing age, subtype of LAA, and higher uric acid levels had prognostic value for impaired CA [4]. However, differences in CA in different stroke subtypes might be attributable to varied pathological changes in cerebral blood vessels and differences in influencing factors, which need to be considered individually. In ischaemic stroke associated with SAO, CA is impaired in both hemispheres rather than in the symptomatic side, and the influencing factors are unclear. It has been shown that penetrating parenchymal arteries and arterioles may contribute to approximately 50% of cerebrovascular resistance, while the remainder is derived from the large extracranial arteries and intracranial pial blood vessels [1]. In terms of the pathological basis of SAO in cerebral blood vessels, it has been hypothesized that a heavier burden of bilateral cerebral small vessel disease (CSVD) may coexist with SAO, which may lead to global impairment of CA in patients with SAO.

In the current study, we explored clinical factors, including CSVD imaging markers and vascular-related risk factors (such as blood pressure management), which may be related to CA impairment in patients with stroke attributed to SAO, to facilitate improved CA after SAO.

## Methods

### Participants

We conducted a prospective study of consecutive patients with ischaemic stroke attributable to SAO who were admitted to the Department of Neurology of the First Affiliated Hospital of Shenzhen University, from September 2017 to April 2019. This study was approved by the Medical Ethics Committee of the First Affiliated Hospital of Shenzhen University. All the participants provided written informed consent.

Patients were included in this study if they (a) were aged between 18 and 80 years; (b) were admitted within 7 days after stroke symptom onset; (c) underwent diffusion-weighted magnetic resonance imaging (DWI) and computed tomography angiography (CTA) and/or digital subtraction angiography (DSA) within 7 days after stroke onset; (d) were classified as having SAO according to the TOAST classification with minor modification; and (e) had a sufficient bilateral temporal bone window for insonation of the middle cerebral arteries (MCA). Patients (a) who had ischaemic stroke attributed to LAA, cardiac embolism, stroke with other determined aetiologies, or stroke with undetermined aetiologies according to the TOAST classification with minor modifications; (b) who had clinical–DWI correlations that did not match the manifestations of acute infarction; (c) with evidence of intracranial or extracranial large arterial stenosis; (d) who had concomitant diseases affecting cerebral haemodynamics (i.e., atrial fibrillation, severe anaemia, hyperthyroidism, or congenital heart disease); (e) who received intravenous thrombolysis and/or endovascular therapy; or (f) who were diagnosed with cancer or mental diseases were excluded.

The following clinical information was collected: age, sex, history of hypertension, diabetes mellitus, ischaemic heart disease, atrial fibrillation, valvular heart disease, current smoking, National Institute of Health Stroke Scale (NIHSS) at admission, blood pressure at admission as measured with a sphygmomanometer, systolic blood pressure (SBP), diastolic blood pressure (DBP) at admission, body mass index (BMI), results of neuroimaging, carotid duplex ultrasonography, 24-h electrocardiography (Holter), 12-lead electrocardiography, and laboratory results for total cholesterol, triglycerides, and homocysteine. We obtained the MAP as the average of the SBP and DBP.

### Brain MRI

The MRI study included T1-weighted images, T2-weighted images, DWI, fluid-attenuated inversion recovery (FLAIR), and susceptibility-weighted images (SWI) obtained using a 1.5-Tsela MR scanner (Siemens, Munich, Germany). We evaluated the presence of four CSVD markers: lacunae, white matter hyperintensities (WMH), microbleeds, and enlarged perivascular spaces (PVS). In addition, we assessed the total CSVD imaging burden scores using the Standards for Reporting Vascular Changes on Neuroimaging (STRIVE) criteria and scale [12, 13].

Lacunae were defined as hypointensities (3–15 mm) on T1-weighted images and hypointensities surrounded by bright rims on FLAIR images. WMH were defined as signal abnormalities of variable size in the white matter that showed the following characteristics: hyperintensity on T2-weighted images, such as FLAIR without cavitation (as compared to cerebrospinal fluid), including periventricular white matter changes and deep white matter changes. Microbleeds were defined as small (generally 2–5 mm, but up to 10 mm in diameter) areas of signal void with associated blooming observed on SWI. The neuroradiologist additionally used a T1-weighted scan to confirm the location of microbleeds and to differentiate these from ventricular calcification or sulcal vessels. PVS was defined as fluid-filled spaces that followed the typical course of a vessel as it traversed the grey or white matter, with signal intensity similar to cerebrospinal fluid on all sequences. The total CSVD burden score was calculated using an ordinal scale ranging from 0 to 4 (1 point was given for each of the four markers examined) [13]. The MRI findings were assessed by two experienced neuroradiologists who were blinded to the patients’ clinical status.

### Cerebral autoregulation protocol

The CA examination protocol was performed according to a white paper from the International Cerebral Autoregulation Research Network [14]. All patients were asked to avoid nicotine, caffeine, alcohol, and all types of sleep medication for at least 24 h before the CA examination. Evaluations were conducted in the stroke unit with the head of the bed at 0° during 5 min of recording. ABP was recorded by placing a finger cuff on the unaffected side using a Finometer (Finometer Model 1, Delica, China). CBF velocity was bilaterally recorded from the M1 segment of the MCA (depth of 50–55 mm) using a transcranial Doppler (TCD). TCD measurements were obtained 7 days after onset using a 2-MHz monitoring probe (Delica) placed on the left and right transtemporal windows for MCA insonation and was maintained for at least 5 min using a fixing device to ensure a constant insonation angle. Transfer function analysis (TFA) was used for CA assessment, as previously described [15, 16]. The results of TFA were attained in the 0.02–0.2 Hz frequency range. The transfer function gain and phase reflect the responses of CBF velocity to blood pressure fluctuations. Gain quantified the damping effect of CA on the magnitude of ABP oscillations. Phase represented the temporal difference between the oscillation transmissions from the mean ABP to the mean CBF velocity.

### Statistical analysis

Continuous variables are presented as median together with the upper (75%) and lower (25%) quartile, and categorical variables are presented as frequencies (percentages). Normality was assessed using the Shapiro-Wilk test. The samples for both bilateral gain (W test: *P* < 0.001) and bilateral phase (W test: *P* = 0.020) were not selected from a normal population, and therefore the Wilcoxon matched-pairs signed-ranks test was adopted when comparing differences in CA parameters (gain and phase) between the affected and unaffected hemispheres. The relationship between clinical factors and CA parameters was assessed using univariate linear regression analysis. Multivariate linear regression analyses were conducted to determine the relationship between the clinical factors and CA parameters (gain and phase). All variables showing statistical significance in the univariate linear regression analysis, as well as some common small vessel risk factors (sex, hypertension, and total CSVD burden score), were included as independent variables in the multivariate linear regression model to identify factors associated with CA. We adjusted for MAP on admission and MAP > 105 mmHg on admission in multivariate models 1 and 2, respectively. All collected data were analysed using SPSS (version 26.0; IBM, Armonk, USA) and R software (the R Foundation, Indianapolis, USA).

## Results

### Demographic and clinical characteristics

Table 1 summarises the baseline characteristics of the patients. Sixty-three patients with ischemic stroke attributed to acute SAO (mean age, 56.3 ± 9.9 years; 87.30% males) were enrolled in the study. Fourteen patients had a history of diabetes mellitus. MAP fluctuated between 76 and 148 mmHg at admission. In 35 patients (55.6%), MAP exceeded 105 mmHg on admission. In terms of the total CSVD burden, 49 patients (77.8%) scored ≥1 point.

**Table 1:** Clinical characteristics of the participants

Characteristics	Total (*n* = 63)
**Vascular risk factors**	
Age (years)	57.0 (48.0, 64.0)
Sex (male), %	55 (87.3)
Hypertension, %	45 (71.4)
Diabetes mellitus, %	14 (22.2)
Current smoking, %	31 (49.2)
BMI	24.2 (22.2, 26.0)
Total cholesterol (mmol/L)	1.4 (1.1, 1.8)
Triglycerides (mmol/L)	3.9 (3.1, 4.8)
Homocysteine (μmol/L)	12.1 (9.7, 14.5)
MAP on admission (mmHg)	106.3 (101.0, 119.0)
MAP > 105 mmHg on admission, %	35 (55.6)
**Brain MRI Markers**	
Lacunae, %	37 (58.7)
Microbleeds, %	20 (31.7)
Perivascular space, %	13 (20.6)
White matter hyperintensities, %	13 (20.6)
Total CSVD burden score (0/1/2/3/4), %	14/30/7/9/3 (22.2/47.6/11.1/14.3/4.8)
**CA parameters**	
Ipsilateral gain, (%/%)	0.6 (0.4, 0.8)
Contralateral gain, (%/%)	0.5 (0.4, 0.7)
Ipsilateral phase, degree	25.8 (13.5, 34.6)
Contralateral phase, degree	25.7 (15.9, 34.7)

Data are presented as median (lower quartile; upper quartile) or absolute number (percentage)

*BMI* Body mass index, *MAP* Mean arterial pressure, *CSVD* Cerebral small vessel disease, *CA* Cerebral autoregulation

Neither gain nor phase CA parameters differed between the ipsilateral and contralateral sides in these patients (*P* = 0.050 and *P* = 0.801, respectively).

### Correlation between cerebral autoregulation and clinical parameters

*P*-values for the univariate analyses of the CA parameters and clinical factors are shown in Supplementary Table 1. Patients with diabetes mellitus had a significantly higher bilateral gain (indicating worse CA) than did patients without diabetes (ipsilateral 0.890%/% vs. 0.564%/%, *P* = 0.006; contralateral 0.760%/% vs. 0.538%/%, *P* = 0.021). Patients who had MAP > 105 mmHg on admission showed a significantly lower bilateral gain (more effective CA) than those who did not (ipsilateral 0.523%/% vs. 0.778%/%, *P* = 0.010; contralateral 0.516%/% vs. 0.677%/%, *P* = 0.045). There was no significant correlation of CA parameters on any side with the following factors: sex, history of hypertension, smoking, BMI, total cholesterol level, triglyceride level, and homocysteine level. No significant correlations were found between CA parameters and CSVD characteristics (neither CSVD markers nor the total CSVD burden score).

The multivariate linear regression models of bilateral CA parameters are summarised in Table 2. In model 1, MAP on admission was significantly associated with bilateral gain (*P* < 0.05). In model 2, multivariable analysis showed that diabetes mellitus was a significant independent predictor of bilateral gain (ipsilateral *P* = 0.015; contralateral *P* = 0.038). Moreover, a MAP > 105 mmHg on admission was a significant predictive value for ipsilateral gain (*P* = 0.019). There was a trend for an association between MAP > 105 mmHg on admission and contralateral gain, although the difference did not reach statistical significance (*P* = 0.069). No factors significantly associated with phase were detected.

**Table 2:** Multivariate linear regression models of CA parameters

Clinical factors	Gain				Phase			
	Ipsilateral		Contralateral		Ipsilateral		Contralateral	
	OR	95% CI	OR	95% CI	OR	95% CI	OR	95% CI
**Model 1**								
Age, years	1.00	0.99–1.01	1.00	0.99–1.01	0. 69	0.46–1.02	0.84	0.57–1.25
Sex (male)	1.20	0.91–1.58	1.16	0.92–1.47	1. 50	0–73,665.66	114.17	0–5,727,688.9
Hypertension	1.24	0.97–1.58	1.13	0.92–1.40	0	0–57.60	0.01	0–187.22
Diabetes mellitus	1.25	1.00–1.56	1.17	0.97–1.41	0.63	0–3498.39	81.05	0.01–460,940.38
Total CSVD burden score	0.96	0.88–1.05	0.98	0.91–1.06	3.01	0.1–89.65	1.96	0.07–58.79
MAP on admission, mmHg	0.99^a^	0.98–1.00	0.99^a^	0.99–1.00	0.93	0.7–1.23	0.90	0.68–1.2
**Model 2**								
Age, years	1.00	1–1.01	1.00	0.99–1.01	0.70	0.48–1.02	0.88	0.60–1.28
Sex (male)	1.25	0.94–1.66	1.19	0.93–1.51	1.78	0–95,380.3	137.52	0–7,720,138.07
Hypertension	1.19	0.93–1.52	1.09	0.89–1.34	0	0–24.58	0.01	0–58.42
Diabetes mellitus	1.32^a^	1.06–1.64	1.22^a^	1.01–1.47	1.00	0–4319.18	159.08	0.04–711,293.88
Total CSVD burden score	0.94	0.86–1.02	0.96	0.90–1.04	2.56	0.09–72.83	1.57	0.05–45.22
MAP > 105 mmHg on admission	0.77^a^	0.63–0.94	0.85	0.72–1.01	0.31	0–643.45	0.25	0–544.41

^a^Significant factors according to multivariate linear regression models

*OR* Odds ratio, *CI* Confidence interval, *CSVD* Cerebral small vessel disease, *MAP* Mean arterial pressure

## Discussion

In the present study, we found that CA was similar in symptomatic and non-symptomatic hemispheres of SAO-associated stroke patients. MAP on admission and diabetes mellitus were both significant independent predictors of CA impairment in ischaemic stroke due to SAO, while our results indicate that CSVD may not be the main factor affecting bilateral CA in patients with SAO.

Impairment of CA after ischaemic stroke has long been a concern. Animal models have demonstrated impaired CA in peri-infarct tissue within 24 h after stroke [17]. The presence or absence of CA in ischaemic stroke has a critical effect on the maintenance of stable blood flow in the ischaemic penumbra and on excessive hyperperfusion. In recent years, it has been reported that impaired CA is associated with various cerebrovascular diseases and that it often indicates a poor prognosis. CA has been shown to vary characteristically among the different stroke subtypes. Using the TFA approach to derive the CA parameters of gain and phase, Immink et al. found that dynamic CA is impaired ipsilaterally in ischaemic stroke in the MCA territory, but bilaterally in ischaemic stroke involving lacunae [9]. Using the same measurement method, another study demonstrated that dynamic CA is impaired ipsilaterally in LAA-associated stroke, but bilaterally in SAO-associated stroke [11]. In our study, we observed similar CA in both hemispheres in patients with SAO-associated stroke, which was consistent with previous reports of bilaterally impaired CA. The factors influencing CA impairment in both hemispheres, rather than only the symptomatic side in SAO-associated stroke warrant further investigation.

Generally, the gain CA parameter (amplitude) quantifies the damping effect of CA on the magnitude of oscillations in blood pressure [1]. Gain is a continuous variable. For example, a value of 0.5 suggests that 50% of the relative amplitude of CBF velocity is attenuated with respect to a unit of change in ABP. Effective CA attenuates gain; thus, a low gain indicates the presence of CA, whereas a high gain indicates diminished effectiveness of the CA. Moreover, a high gain indicates that a greater relative amplitude of CBF velocity is attenuated with fluctuations in blood pressure, suggesting that the distal arterioles and capillaries do not respond to changes in blood pressure. Data from previous studies have shown that impaired CA is likely associated with the loss of endothelial and smooth muscle function [2]. Thus, in stroke due to SAO, functional and structural changes in the small vessels may play a role in global cerebral haemodynamic impairment.

However, it remains unclear whether the globally impaired CA in SAO is caused by acute infarction or chronic small-vessel disease. Existing hypotheses tend to attribute it to bilateral small vessel disease. CSVD is associated with changes in cerebral small vessels and consequent brain damage in the white and grey matter [18]. A recent study in patients with CSVD demonstrated that CA was compromised, and some specific neuroimaging characteristics (total CSVD burden, white matter hyperintensities, severe PVS, and lobar cerebral microbleeds) might indicate more severe CA impairment [19]. Another study in patients with Alzheimer’s disease demonstrated an interrelationship between Alzheimer’s disease pathology, CSVD, and CA [20]. However, in the present study in SAO-associated stroke patients, no significant correlations were found between CA parameters and CSVD characteristics (neither CSVD markers nor the total CSVD burden score). Nonetheless, it is worth noting that diabetes mellitus, a common risk factor for small-vessel disease, was significantly associated with impaired CA. This suggests that globally impaired CA in patients with SAO may be induced by acute stimulation of vascular events based on extensive chronic small vessel dysfunction. More robust longitudinal studies are needed to investigate the mechanism underlying bilateral CA impairment. Controlling such risk factors affecting cerebral small vessels may be a feasible method for protecting against CA.

To date, few studies have explored the risk factors of CA impairment in ischaemic stroke. Recently, one study that enrolled 67 stroke patients regardless of their etiological subtypes suggested that increasing age, subtype of LAA, and higher uric acid levels had prognostic value in terms of disturbed autoregulation [4]. In the present study, we found that MAP on admission and diabetes mellitus were significant independent predictors of CA impairment in ischaemic stroke patients with SAO. These inconsistent results may be attributed to the varied stroke aetiology among the patients enrolled in the two studies. Considering the different subtypes of stroke with highly variable features, exploring CA characteristics on an individual etiological basis in this population is imperative.

Following ischaemic stroke, a significant number of patients present with hypertension, and some trials have tested approaches for blood pressure management in the acute and subacute stages [21–23]. Because of the complexity of CA, significant controversies exist regarding blood pressure management after ischaemic stroke. The ideal blood pressure range after ischaemic stroke is unknown. Current guidelines recommend permissive hypertension after an ischaemic stroke. In patients who do not receive thrombolysis, it is recommended that blood pressure treatment should be avoided unless SBP > 220 mmHg or DBP > 120 mmHg within the initial 24 h after stroke [24]. However, in the era of precision medicine, an optimal blood pressure range should probably depend on individual CA variability, temporal and spatial heterogeneity of stroke pathophysiology, and stroke subtype.

Currently, the blood pressure control strategy for patients with SAO is not conclusive. In this study, we found that a low MAP on admission was a significant independent predictor of CA impairment in ischaemic stroke due to SAO, and an appropriate increase in MAP (likely MAP > 105 mmHg) in the acute phase after stroke may help to improve CA. This is consistent with the suggestion of permissive hypertension after an ischaemic stroke. Our results suggest that CA in patients with SAO may be more sensitive to low blood pressure and hypoperfusion injury. In ischaemic stroke, there is an assumption that CA would be impaired, and that CBF would need to be maintained within safe limits by controlling ABP. In patients with Moyamoya disease, CBF was found to be susceptible to small blood pressure changes, and that CA might be affected by short dynamic blood pressure modifications [25]. Thus, an increase in MAP could be hypothesised to serve the purpose of maintaining CBF (specifically in the penumbra), and lowering MAP may therefore be disadvantageous. Our results helped to establish a relatively ideal blood pressure range following ischaemic stroke with SAO. However, more precise CA-oriented blood pressure management in SAO patients still needs to be established and verified by sufficient preclinical and clinical evidence in a future study.

This study had several limitations. First, in stroke patients with sufficient temporal bone windows for insonation of the MCA in routine TCD ultrasonography, the successful rate of TCD monitoring at our lab was more than 90%. However, female tended to have lower stroke incidence and poor bilateral temporal windows that failed to complete TCD monitoring. Therefore, there were fewer female participants in this study. Second, post-stroke changes in CA and MAP over time were not evaluated. Third, end tidal CO2 was not recorded. Finally, the sample size was small. Large-sample multicentre studies are needed in the future to provide more evidence in this regard.

## Conclusions

In ischaemic stroke due to SAO, patients with MAP > 105 mmHg on admission tended to have a better ipsilateral CA. Diabetes mellitus appears to be an independent risk factor for CA impairment in patients with SAO-associated stroke. CSVD may not be the main factor affecting bilateral CA in patients with SAO.

## Supplementary Information

**Additional file 1: Supplementary Table 1.** Univariate analyses of ipsilateral CA parameters and clinical factors.

## Data Availability

All data generated or analyzed during this study are included in this published article and its supplementary information files.

## Abbreviations

ABP: Arterial blood pressure
CA: Cerebral autoregulation
CBF: Cerebral blood flow
CSVD: Cerebral small vessel disease
LAA: Large artery atherosclerosis
MAP: Mean arterial pressure
SAO: Small artery occlusion
