# Supplementary material for: Clinical factors associated with cerebral autoregulation in ischemic stroke related to small artery occlusion
Source: BMC Neurol. 2022 Sep 22;22:364. doi: 10.1186/s12883-022-02854-4 (PMC9494772; doi:10.1186/s12883-022-02854-4)
Supplement: Supplementary file 1 — Additional file 1: Supplementary Table 1. Univariate analyses of ipsilateral CA parameters and clinical factors. [file 12883_2022_2854_MOESM1_ESM.docx]

**Supplementary Table 1. Univariate analyses of ipsilateral CA parameters and clinical factors**

| Clinical factors |  | | Ipsilateral gain | |  | | Ipsilateral phase | |
| --- | --- | --- | --- | --- | --- | --- | --- | --- |
|  | OR | 95% CI | | *P* value | OR | 95% CI | | *P* value |
| Age, years | 1.01 | 1.00 - 1.02 | | 0.080 | 0.69 | 0.49 - 0.96 | | 0.032 |
| Sex (male) | 1.08 | 0.80 - 1.45 | | 0.616 | 88.03 | 0 - 2658444.35 | | 0.398 |
| Hypertension | 1.06 | 0.85 - 1.31 | | 0.633 | 0 | 0 -1.29 | | 0.063 |
| Diabetes mellitus | 1.38 | 1.11 - 1.73 | | 0.006 | 0.14 | 0 - 576.52 | | 0.648 |
| Smoking | 1.09 | 0.89 - 1.32 | | 0.412 | 0 | 0 - 1.25 | | 0.063 |
| BMI | 0.98 | 0.94 - 1.01 | | 0.184 | 1.54 | 0.44 - 5.37 | | 0.501 |
| Total cholesterol, mmol/L | 0.91 | 0.81 - 1.02 | | 0.104 | 0.32 | 0.01 - 17.04 | | 0.574 |
| Triglycerides, mmol/L | 0.99 | 0.91 - 1.08 | | 0.852 | 0.89 | 0.04 - 18.19 | | 0.939 |
| Homocysteine, µmol/L | 1.00 | 0.99 - 1.01 | | 0.823 | 0.93 | 0.61 - 1.41 | | 0.725 |
| MAP on admission, mmHg | 0.99 | 0.98 - 1.00 | | 0.001 | 0.97 | 0.77 - 1.22 | | 0.775 |
| MAP > 105 mmHg on admission | 0.77 | 0.64 - 0.94 | | 0.010 | 0.49 | 0 - 515.24 | | 0.843 |
| Lacunae | 0.90 | 0.74 -1.10 | | 0.295 | 1.89 | 0 - 2110.90 | | 0.859 |
| Microbleeds | 0.98 | 0.79 - 1.21 | | 0.854 | 0.39 | 0 - 641.61 | | 0.802 |
| Enlarged perivascular space | 0.83 | 0.65 - 1.05 | | 0.126 | 28.74 | 0.01 - 140666.05 | | 0.441 |
| White matter hyperintensities | 1.08 | 0.84 - 1.37 | | 0.554 | 0 | 0 - 7.13 | | 0.139 |
| Total CSVD burden score | 0.96 | 0.88 -1.05 | | 0.379 | 0.64 | 0.03 - 14.31 | | 0.778 |

OR, odds ratio; CI, confidence interval; MAP, mean arterial pressure; CSVD, Cerebral small vessel disease.
